# Prevalence of malocclusion and assessment of treatment needs in β-thalassemia major children

**DOI:** 10.1186/s40510-016-0120-6

**Published:** 2016-03-09

**Authors:** Deepak Kumar Gupta, Satinder Pal Singh, Ashok Utreja, Sanjeev Verma

**Affiliations:** Department of Orthodontics and Dentofacial Orthpaedics, Dr Harvansh Singh Judge Institute of Dental Sciences and Hospital, Panjab University, Chandigarh, India; Unit of Orthodontics, Oral Health Sciences Centre, PGIMER, Chandigarh, India

**Keywords:** Prevalence, Thalassemia, TPI index, Malocclusion

## Abstract

**Background:**

The objective of this study is to evaluate the prevalence of malocclusion and treatment needs in transfusion dependent β-thalassemia major children.

**Methods:**

One hundred transfusion dependent β-thalassemia major children visiting the Department of Pediatrics were selected randomly and evaluated for malocclusion with Angle’s classification and Dewey’s modification. The orthodontic treatment needs were also assessed using Grainger’s treatment priority index (TPI). The orthodontic treatment needs were compared to normal children.

**Results:**

The assessment of treatment needs revealed a higher prevalence of handicapping and severely handicapping malocclusion in thalassemic children compared to normal children. The thalassemic patients were found to show significantly more Angle’s Class II malocclusion (55 % vs. 15.7 %) when compared to normal children.

**Conclusions:**

The higher prevalence of Angle’s Class II malocclusion and definitive malocclusion in thalassemic children indicates the importance of preventive orthodontic procedures and efforts towards providing orthodontic treatment to these children.

## Background

Orthodontics is the discipline within the framework of dentistry which has for its purpose the diagnosis and treatment of dentofacial abnormalities. This twofold purpose is best expressed in those children who either exhibit a dentition of extreme degree of malocclusion or facial features of marked variations. When a child exhibits both extremes, he deserves a thorough study of his affliction. A child with thalassemia major (Cooley’s anemia) falls in such group [1].

Thalassemia is one of the commonest genetic disorders known to mankind. Thalassemia and other hemoglobinopathies are widespread globally. Thalassemia may have originated 50,000 years ago in a valley South of Italy and Greece covered by Mediterranean Sea. The name thalassemia has been derived from a Greek word “Thallus” meaning sea. Thalassemia was recognized as a clinical entity by Cooley and Lee. Thomas B. Cooley (1927), a pioneer pediatrician, reported seven cases of splenomegaly with anemia, peculiar bone changes, and characteristic facies [2]. Originally described as a separate disease entity, this disease is now known as Cooley’s anemia or β-thalassemia major. The term “thalassemia” refers to a group of blood disorders characterized by decreased synthesis of one of the two types of polypeptide chain (α or β) which form the normal adult hemoglobin molecule (HbA, α_2_ β_2_), resulting in decreased filling of the red cells with hemoglobin and anemia. Based on the genetic and clinical entities, thalassemia is classified as homozygous, heterozygous, or compound heterozygous [3]. The homozygous form (thalassemia major) exhibits most severe clinical symptoms with marked orofacial defects. The inheritance of beta thalassemia is an autosomal recessive disorder with the chromosomal abnormality on short arm of 11th chromosome (chromosome # 11p15.5).

The suggested causes of growth retardation and maturation of the skeleton in thalassemic major patients include chronic anemia, hyperparathyroidism, and somatomedin deficiency, a factor that stimulates cartilage growth [4–6]. The dentition shows protrusion, flaring, and spacing of the anterior teeth, open bite, and other degrees of malocclusion [7]. The tooth crown size and crown length in thalassemic subjects are significantly smaller than those in the unaffected groups [8, 9]. The literature shows increased prevalence and severity of malocclusion (dentional and craniofacial abnormalities) in thalassemic patients [8, 9]. Hattab FN^1^ observed increased overjet in 25.9 % of the patients and more than half of the patients exhibited frontal bossing, saddle nose, and to less extent maxillary protrusion, giving in severe cases (16.7 %) a “chipmunk”-like appearance [10]. The prevalence and severity of malocclusion is also dependent on the medical treatment provided to the thalassemic children [11]. Patients with craniofacial anomalies often have appearance concerns and related social anxiety which can affect their quality of life. Singh and Moss in their study assessed the psychological impact of facial and dental appearance in patients with craniofacial anomalies in comparison to a general population control group [12]. It is thus important to study the prevalence and orthodontic treatment needs in our population that may be different from other groups. The treatment priority index (TPI) index was used in the study because of its simple and efficient application  in epidemiological surveys of malocclusions without undue cost and energy [13].

Thus, the aim of the present study was to evaluate the prevalence of the type of malocclusion and to assess orthodontic treatment needs in the thalassemic children.

The study was carried out with the following aims and objectives:

1. To evaluate the prevalence of malocclusion in transfusion-dependent beta thalassemia children using Angle’s classification and Dewey’s modification
2. To assess the orthodontic treatment needs using Grainger’s TPI

## Methods

The study was conducted at the Unit of Orthodontics, Oral Health Science Centre in collaboration with Thalassemia ward at Department of Pediatrics, Advanced Pediatric Centre (APC), Postgraduate Institute of Medical Education and Research (PGIMER), Chandigarh. Appropriate ethical clearance was granted to conduct the study by the board of studies of the institution. The study comprised of one hundred transfusion-dependent beta thalassemia major children (71 males, 29 females) between the age range of 12–17 years. The mean age of sample was 13.84 years. The children were selected on a random basis from the patient regularly coming for transfusion in the thalassemic ward at APC, PGIMER, Chandigarh. The first 100 beta thalassemia patients visiting the department in the defined age group were assigned to the examiner for evaluation of type of malocclusion and TPI index, without the examiner’s choice. The examiner was trained and standardized in the use of TPI. The intraoral examination of all the patients was done and recorded by the same examiner.

### Selection criteria

The patients included in this study fulfilled the following criteria:

(i)  Diagnosed case of homozygous beta thalassemia (thalassemia major), receiving regular blood transfusion.
(ii)  Patients 12–17 years of age.
(iii)  Patient who had not undergone any kind of orthodontic treatment.
(iv)  Patient not affected by conditions like gross physical or mental disability.
(v)  The first 100 patients visiting the department regularly for transfusion every month were evaluated. They constituted the major portion of the patients in the concerned age group visiting the thalassemia clinic for regular transfusion.

The control group comprised a proportioned sample from epidemiological survey of prevalence of malocclusion in 12–17 years school children; 500 students in the defined age group were examined randomly in the two different schools in the vicinity without any priority, and the data was recorded.

A Performa was used to record the various aspects of malocclusion and to record the various parameters of Grainger’s TPI index [14]. The Performa (TPI data collection form) was designed to include patient details, type of malocclusion i.e. Angle’s classification, and parameters of TPI index (first molar relation, horizontal and vertical incisor relation, tooth displacement score, congenitally missing incisors, posterior crossbite, and any other defect.

A total of TPI scores were calculated, and the level of severity was assessed according to malocclusion severity estimate. Levels of severity of a malocclusion as established by the malocclusion severity estimate (MSE) and used for the present study are given below:

**Table:** 

I. Virtually classic normal occlusion	0
II. Minor manifestations of malocclusion and treatment need is slight	1–3
III. Definite malocclusion, but treatment elective	4–6
IV. Severe handicap, treatment highly desirable	7–9
V. Very severe handicap with treatment mandatory	>10

### Statistical analysis

#### Intraexaminer and interexaminer variability and reproducibility

The observations in TPI index of approximately 10 % of the sample were graded after an interval of 1 week to standardize the investigator for a reproducible result. The investigator was trained for evaluation using TPI index. Further, the observations of 10 % patients were also graded by one of the experienced examiners so as to test the interexaminer reproducibility which was found to be satisfactory.

The means and percentages were calculated for the different population groups. The comparison of population means was done using “Z” test to compare the prevalence of malocclusion and malocclusion traits between the two groups under study.

## Results

### Prevalence of malocclusion

The results in the present study show that significantly less (*p* < 0.001) thalassemic children have Angle’s Class I malocclusion (44.0 % vs 82.2 %) than normal children. The thalassemic patients show significantly more Angle’s Class II malocclusion (55 % vs 15.7 %) when compared to normal children (Table 1).

**Table 1:** Comparison of prevalence of Class I, Class II, and Class III malocclusion in thalassemic and normal children

	Angle’s Class I Dewey’s modification								Angle’s Class II					Angle’s Class III			
	Class I type 1 (%)	Class I type 2 (%)	Class 1 type 3 (%)	Class I type 4 (%)	Class I type 5 (%)	Bimax (%)	Spacing (%)	Total (%)	Class II Div 1 (%)	Class II Div 2 (%)	Class II subDiv 1 (%)	Class II subDiv 2 (%)	Total (%)	Type 1 (%)	Type 2 (%)	Type 3 (%)	Total (%)
Thalassemic children	25	8	0	2	0	5	4	44	34	5	15	1	55	0	0	1	1
Normal children	69	9	0.4	0.9	0.59	8	12	82.2	6	3.69	4	2	15.69	0.6	0.49	0.4	1.49

Further within the Class I malocclusion group, Class I type 1 malocclusion was significantly less (*p* < 0.001) in thalassemic children when compared to normal (25 % vs 69.6 %).

In the Class II subgroup, Class II div 1 malocclusion shows the maximum occurrence.

The prevalence of Class III malocclusion is less than the normal population with only one case reported in the present study (Table 1).

### Assessment of overjet, prevalence of tooth displacement, and buccal and lingual crossbite

In thalassemic children, a positive overjet was found in 99.0 % of patients and it was not different from normal population (98.5 %). None of the thalassemic patients showed overjet of 1 mm compared to 3.9 % of normal children having overjet of 1 mm (*p* < 0.05). The prevalence of tooth displacement is more in thalassemic patients. The prevalence of buccal and lingual crossbite is also more in thalassemic patients (Table 2).

**Table 2:** Comparison of prevalence of tooth displacement, buccal and lingual crossbite, and overjet in thalassemic and normal children

	Tooth displacement					Buccal crossbite		Lingual crossbite		Overjet				
	1–2	3–4	5–6	7–8	>9	Single tooth (%)	Total prevalence (%)	Single tooth (%)	Total prevalence (%)	1	2–4	5–6	7–8	>9
Thalassemic children	8	30	42	10	10	17	20	17	21	0	48	32	14	6
Normal children	30	18	8	5	2	2	3	3	4	4	83	8	4	1

### Treatment needs assessment

TPI scores showed normal occlusion (TPI score 0) to be significantly less (*p* < 0.001) in thalassemic children when compared to normal children. On the other hand, definite malocclusion (TPI scores 4–6.99) was significantly more in thalassemic children compared to normal children. Similarly handicapping malocclusion (TPI scores 7–9.9) and severely handicapping malocclusion (TPI scores >10) was significantly more (*p* < 0.05) prevalent in thalassemic children than normal children (Table 3).

**Table 3:** Comparison of TPI scores in thalassemic and normal children

TPI scores	Normal occlusion 0 (%)	Minor malocclusion 1–3.99 (%)	Definite malocclusion 4–6.99 (%)	Handicapping malocclusion 7–9.99 (%)	Severely handicapping malocclusion >10.0 (%)
Thalassemic children	6	26	36	18	14
Normal children	40	37	10	5	3

The comparison of TPI score of thalassemic children in the present study shows 32 % in the no or minor malocclusion groups, 36 % in the definite malocclusion group, and 32 % in the handicapping and severely handicapping malocclusion group.

Similar comparison of TPI score of normal children in the present study shows 77 % in the no or minor malocclusion groups, 10 % in the definite malocclusion group and 8 % in the handicapping and severely handicapping malocclusion group.

## Discussion

The prevalence and severity of malocclusions in transfusion-dependent thalassemic patients of the age group 12–17 years  was done as this is a common age when orthodontic treatment is sought. The TPI was selected for this evaluation as it has proved to be a useful epidemiological indicator of malocclusion [15]. It has been found to be highly reproducible and valid [16]. Application of the TPI is practicable and requires less clerical time when compared with the Occlusal Index (OI) [17]. Thus, the TPI is a useful index for measuring need for treatment and as an aid in the identification of children who can benefit most from orthodontic treatment [14].

The present study reported a ratio of 1:1.25:0.01 between angle Classes I, II, and III malocclusion, respectively, in the thalassemia group which contrasted to a normal population ratio of 5.2:1:0.01. Pusaksrikit et al. reported the ratio between various angle classes, i.e., I, II, III as 2.7:1:0 which contrasted to a normal population pattern of 3:1:1 [18].

Giuseppina in a study reported Class I, II, III malocclusions, and asymmetries in 40.4, 29.2, 3.2 and 27.1 % of the sample, respectively. The objective need for orthodontic treatment (grades 4 and 5 of IOTN) was registered in 1077 subjects (41.2 %) [19].

The higher prevalence of Class II malocclusion in thalassemic children in the past has been attributed both to marrow hyperplasia occurring due to chronic anemia, resulting in maxillary prominence and also due to mandibular retrusion occurring because of generalized growth retardation in the thalassemic children by different authors [20–23].

In the present study, the assessment of malocclusion using TPI scores in normal population shows no or minor malocclusion in 77 % cases. This is similar to results of studies by Bhardwaj et al., Shivakumar et al., Esa et al., and van Wyk et al. These studies using Dental Aesthetic Index scores show no or minor malocclusion in the range of 60 to 80 % in the normal population [24–27]. Ugur et al. conducted a study to evaluate the prevalence of malocclusion and to assess the need for orthodontic treatment among 6–10-year-old Turkish primary school children. The TPI was used to record and measure the malocclusions; 40.38 % of the observed population showed normal occlusion, 21.85 % had minor manifestations of malocclusion and treatment need was slight, 25.17 % of the subjects showed definite malocclusion, 7.54 % had severe malocclusion, and 5.06 % had a very severe handicap with a mandatory treatment requirement. Thus, according to this study, there is definite need for orthodontic treatment in 37 % of individuals [28].

The present study indicates that 68 % of the thalassemic patients definitely need orthodontic treatment which is 18 % for normal population. Comparison with any of the previous studies show that the prevalence of malocclusion in thalassemic patients is also much more than that in the normal population. The assessment of treatment needs revealed a higher prevalence of handicapping and severely handicapping malocclusion in thalassemic patients compared to normal children. This necessitates the need for providing orthodontic treatment to these children.

In the present study, occurrence of Class III malocclusion was an interesting observation. It is difficult to explain this observation as there was no familial history and literature does not support the occurrence of Class III in thalassemic patients.

The present study was conducted in an environment where the patients are regularly transfused and transfusion is started at very early stages. The blood hemoglobin levels are regularly monitored and maintained. This decreases the extent of malocclusion. Thus, we may expect more severe malocclusions in other environments where the facilities for these patients are not very favorable. This may further increases the need for orthodontic treatment.

## Conclusions

- The prevalence of Class II malocclusion is higher in thalassemic children.
- Overall definite malocclusion, handicapping malocclusion, and severely handicapping malocclusion is more in thalassemic children than that in normal children, so our efforts should be directed towards providing orthodontic treatment to these children.
- Further, the advancement of medical treatment modalities has led to an increase in life expectancy of these children and demand for orthodontic treatment may increase in future.
- These results underscore the high percentage of orthodontic treatment need in thalassemic patients and indicate the importance of preventive orthodontic procedures.

Since the sample size of the thalassemic group in the study was not very large, further studies may be conducted to confirm the results of the present study. The baseline information outlined in the present study can be appropriately utilized for the future planning to meet the orthodontic treatment need among the population.
